# Preventing Nipple-Areola Complex Ischemia in Reduction Mammaplasty and Mastopexy: A Narrative Review of Risk Factors, Perfusion Optimization, and Early Salvage

**DOI:** 10.7759/cureus.116787

**Published:** 2026-09-23

**Authors:** Gisela Hobson Pontes, Luiz Augusto Sousa Oliveira, Camila Araújo Gomes, Igo Reichmann Losso, Ligia Helena Mendes, Nélio Nunes Cabette Filho

**Affiliations:** 1 Plastic Surgery, Hospital Niterói D'Or, Serviço de Pós-Graduação em Cirurgia Plástica Prof. Ronaldo Pontes, Niterói, BRA; 2 General Surgery, Fundação Hospitalar Santa Terezinha de Erechim (FHSTE), Erechim, BRA

**Keywords:** indocyanine green angiography, ischemia, mastopexy, necrosis, nipple-areola complex, reduction mammaplasty, surgical flaps, tissue perfusion

## Abstract

Nipple-areola complex (NAC) ischemia is among the most feared complications of reduction mammaplasty and mastopexy, ranging from transient venous congestion and partial epidermolysis to full-thickness necrosis with lasting aesthetic and psychological sequelae. Because the transposed NAC depends entirely on the perfusion of its dermoglandular pedicle, viability is determined by a chain of anatomical, patient-related, technical, and systemic physiologic factors, many of which are modifiable. This narrative review synthesizes the contemporary evidence on preventing NAC ischemia across the perioperative continuum, drawing on a focused search of PubMed/MEDLINE and screening of the reference lists of key articles. We summarize the vascular anatomy that underpins pedicle design; the influence of pedicle selection, pedicle length, and resection weight; and the patient-related predictors of compromise, including obesity, macromastia, a long sternal notch-to-nipple distance, and smoking. We then examine modifiable perioperative measures: structured smoking cessation, maintenance of normothermia, meticulous hemostasis, and the limited role of tranexamic acid in preventing the hematoma that can strangle a marginal pedicle, and risk-stratified thromboprophylaxis as part of global circulatory optimization. We appraise intraoperative perfusion assessment, in which clinical judgment is complemented by indocyanine green angiography, and the intraoperative decision to convert to a free nipple graft in extreme cases. Finally, we outline the recognition and stepwise salvage of the compromised NAC, prioritizing early relief of tension and pressure and pharmacologic decongestive and vasodilatory support before ischemia becomes irreversible. Systemic, often-overlooked determinants such as normothermia and thromboprophylaxis are integrated into a practical prevention framework, while we acknowledge that the evidence for some measures, notably the avoidance of external compression, remains largely physiologic and observational.

## Introduction and background

Reduction mammaplasty and mastopexy are among the most frequently performed and satisfying operations in aesthetic and reconstructive breast surgery. In the great majority of techniques, the nipple and areola are carried to their new position on a dermoglandular pedicle rather than being removed and replaced, which makes NAC viability directly and unavoidably dependent on the perfusion of that pedicle. Compromise of this perfusion produces a clinical spectrum that extends from transient hypoperfusion and partial-thickness epidermolysis to irreversible full-thickness necrosis of the nipple and areola, an outcome whose aesthetic and psychological consequences are durable and difficult to correct [1].

Population-level and single-center series characterize complete, full-thickness NAC necrosis as an uncommon but clinically important event, more likely after large-volume resections and in patients with metabolic and vascular comorbidity, whereas partial-thickness changes and reversible venous congestion are appreciably more frequent; the absence of standardized definitions for partial versus complete necrosis, however, makes precise incidence estimates difficult and accounts for much of the variation in reported rates [1,2]. Its low baseline frequency is precisely what makes prevention, rather than treatment, the dominant priority: because established necrosis is difficult to reverse and the salvage literature is largely observational, the greatest protection comes from identifying and controlling the determinants of pedicle perfusion before ischemia occurs.

Much of the existing literature addresses the management of established NAC necrosis once it has declared itself [3], whereas the determinants of perfusion are dispersed across anatomical, technical, and perioperative sources that are rarely integrated into a single preventive framework. The purpose of this narrative review is to consolidate that evidence - anatomical, patient-related, technical, and systemic - into a coherent and practically oriented account of how NAC ischemia can be prevented, how perfusion can be assessed intraoperatively, and how an early compromise can be salvaged. In doing so, we deliberately incorporate modifiable systemic factors, such as perioperative normothermia and risk-stratified thromboprophylaxis, that are well established in general and plastic surgical practice but are seldom discussed in the NAC-specific literature.

## Review

Search strategy and study selection

This is a narrative, non-systematic review. We searched PubMed/MEDLINE for English-language articles on nipple-areola complex (NAC) perfusion and ischemia in reduction mammaplasty and mastopexy, using combinations of terms including "nipple-areola complex", "reduction mammaplasty", "mastopexy", "ischemia", "necrosis", "perfusion", "pedicle", "indocyanine green angiography", "tranexamic acid", "normothermia", and "salvage". The reference lists of key articles were screened to identify additional relevant sources, and the bibliographic details and digital object identifiers of all cited references were verified against Crossref and PubMed. No date restriction was applied (coverage through 2026); seminal anatomical and surgical-technique studies were retained regardless of age, while recent evidence was included where available. Consistent with a narrative review, study selection was based on relevance and perceived methodological quality at the authors' discretion; no registered protocol, predefined inclusion or exclusion criteria, or formal risk-of-bias assessment was used.

Vascular anatomy underlying pedicle design

The blood supply of the NAC derives from a redundant anastomotic network fed by perforators of the internal thoracic (internal mammary) artery, branches of the lateral thoracic artery, and the anterior and lateral intercostal perforators, with important contributions from the subdermal plexus and the underlying parenchyma [4]. Detailed anatomical dissection has shown both the richness and the variability of these sources and, critically for the surgeon, that no single pedicle captures all of them; each pedicle design samples a different subset of the network [4,5]. Microdissection studies comparing NAC-bearing pedicles have demonstrated meaningful differences in their vascular reliability, providing an anatomical rationale for pedicle choice rather than mere convention [5]. Contemporary imaging supports these findings in vivo: duplex ultrasound of the female breast confirms a dominant medial and superomedial arterial contribution to the NAC, consistent with the reliability of medially based pedicles [6].

Pedicle selection, pedicle length, and resection weight

In practice, the NAC is perfused through a chosen pedicle, whether inferior, superomedial, medial, central, or superior, whose base incorporates the nourishing vessels. The inferior pedicle, based on a broad dermoglandular column, became popular for its reliability and reproducibility across a wide range of breast sizes [7], while the superomedial pedicle is designed to deliver a robust and consistent supply to the NAC with a favorable arc of rotation during transposition [8]. Pedicle geometry, more than the absolute amount of tissue removed, governs perfusion: excessive pedicle length, acute angulation, and closing tension push the flap toward the limit of its vascular territory. Reassuringly, when a well-vascularized design is used, increasing resection weight does not by itself appear to increase the rate of nipple necrosis, and a large single-technique series of superomedial pedicle reductions found no such relationship [9]. Nevertheless, the anatomical situation created by severe macromastia, namely a very long pedicle carrying the NAC across a great distance, remains the principal setting in which perfusion becomes marginal, and it is here that the surgeon must weigh pedicle-based transposition against primary free nipple grafting [10].

Patient-related risk factors

Risk factors for NAC compromise can be grouped as patient-related and technique-related. Among patient factors, obesity and large resection weight are consistently associated with higher rates of wound-healing complications and NAC compromise; an analysis of 4,545 reduction mammaplasties from the National Surgical Quality Improvement Program (NSQIP) database identified obesity as an independent predictor of early complications [2], and single-center series report a similar gradient [1]. Breast size and geometry matter independently of weight: a long sternal notch-to-nipple distance, the clinical signature of severe ptosis and macromastia, has been linked to higher complication rates and, together with resection-weight asymmetry, to poorer patient-reported outcomes [11]. Smoking deserves particular emphasis as the most important modifiable patient factor: nicotine and the other constituents of cigarette smoke produce cutaneous vasoconstriction and microvascular injury that markedly increase the risk of flap and full-thickness graft necrosis [12]. Nutritional status is a further and easily overlooked modifiable determinant. Deficiency states, whether from restrictive or poorly planned vegetarian or vegan diets, post-bariatric malabsorption, or chronic illness, can impair wound healing and lower the tolerance of marginally perfused tissue, a contribution that may declare itself later in the postoperative course rather than as immediate perfusion failure; preoperative screening and correction of anemia and of protein and micronutrient (iron, vitamin B12, zinc) deficiency is therefore a low-cost adjunct to the measures above [13]. Recognizing which of these factors are fixed, such as breast geometry and established comorbidity, and which are modifiable, such as smoking and glycemic and weight optimization, allows the surgeon to stratify risk and to individualize both technique and perioperative preparation.

Preoperative optimization: smoking cessation

Because smoking is both prevalent and modifiable, structured preoperative cessation is one of the highest-yield preventive interventions available. A systematic review and meta-analysis found that smoking cessation reduces postoperative complications, with a greater benefit as the smoke-free interval before surgery lengthens [14]. Cessation is not merely advice to be given in passing: a systematic review of preoperative smoking-cessation interventions demonstrated that structured programs combining behavioral support with nicotine replacement significantly increase the proportion of patients who actually stop and reduce postoperative morbidity [15]. For elective breast reduction and mastopexy, which are rarely time-critical, this evidence supports a firm expectation of cessation several weeks before surgery, reinforced by an organized cessation pathway rather than an unsupported recommendation.

Intraoperative physiology: normothermia

Maintenance of perioperative normothermia is a systemic, modifiable factor with a direct bearing on tissue perfusion. Hypothermia triggers thermoregulatory cutaneous vasoconstriction, reduces peripheral oxygen delivery, impairs wound healing, and promotes coagulopathy; conversely, keeping patients normothermic reduces surgical-site infection and shortens hospitalization [16]. These mechanisms are directly relevant to a marginally perfused dermoglandular pedicle, in which any further reduction in cutaneous blood flow may be decisive. In a dedicated review of perioperative hypothermia in medium- and large-volume plastic surgery, we previously synthesized these clinical and surgical implications and set out practical prevention strategies, including preoperative and intraoperative active warming, warmed intravenous and irrigation fluids, and continuous temperature monitoring, that are readily applicable to mammaplasty and plausibly favorable to NAC perfusion [17]. Normothermia is thus a low-cost measure that belongs to the global optimization of the microcirculation rather than to infection control alone.

Hemostasis, hematoma, and tranexamic acid

A postoperative hematoma is a mechanical as well as a hemorrhagic problem: an expanding collection beneath a transposed NAC raises local pressure, obstructs venous outflow, and can convert a marginally perfused pedicle into an ischemic one. Meticulous intraoperative hemostasis is therefore part of NAC protection. Interest in tranexamic acid (TXA) as an adjunct to reduce bleeding and hematoma in reduction mammaplasty has grown rapidly, and a systematic review and meta-analysis suggests that it reduces ecchymosis and drain output [18]. Honesty about the limits of this evidence is important, however: a double-blind randomized controlled trial found that topical TXA did not reduce hematoma formation after reduction mammaplasty [19]. The pragmatic conclusion is that TXA may have a role in reducing bleeding but should not be regarded as a substitute for scrupulous surgical hemostasis, which remains the primary defense against the hematoma-driven pathway to NAC compromise.

Global circulatory optimization: thromboprophylaxis

Risk-stratified thromboprophylaxis belongs to the same philosophy of perioperative circulatory optimization. Its indication must be individualized and guided by validated risk assessment, using models that weight obesity, immobility, prior thromboembolism, hormonal therapy, and the magnitude of the procedure, because prophylaxis must be balanced against the bleeding and hematoma risk that itself threatens the NAC [20]. In a narrative review of thromboprophylaxis in intermediate and major plastic surgery, we proposed an integrated decision-making model that links individual risk assessment to the rational selection of mechanical and pharmacologic measures [21]. It must be stated plainly that thromboprophylaxis is aimed at preventing venous thromboembolism and that there is no evidence it directly reduces NAC ischemia; it is discussed here because it is part of a coherent perioperative program directed at circulatory homeostasis and at avoiding complications, such as hematoma, that aggravate pedicle compromise. This candor about mechanism is deliberate: not every systemic measure acts on the NAC directly, and conflating them would misrepresent the evidence. Thromboprophylaxis is therefore included here only as a peripheral component of global perioperative optimization, not as a direct determinant of NAC perfusion.

Intraoperative perfusion assessment

Vigilance begins intraoperatively. Clinical assessment, including color, capillary refill, dermal bleeding on scratch or needle, and turgor of the NAC after transposition, remains the fundamental and always-available method, and the intraoperative recognition of a poorly perfused NAC is the single most important opportunity to change the operative plan. Indocyanine green (ICG) angiography adds objective, quantifiable information to this judgment. Validated first for predicting mastectomy skin-flap necrosis, ICG angiography maps areas of hypoperfusion that are not reliably apparent to the eye [22], and it has been applied specifically to the NAC during breast reduction, where it demonstrates nipple-areola vascularity and can inform intraoperative decisions [23]. More recent work using laser-assisted ICG angiography has extended this assessment to aesthetic reduction mammaplasty [24], and a prospective study of perfusion control during reduction for gigantomastia illustrates how intraoperative ICG can guide the management of the highest-risk NACs [25]. When perfusion is judged inadequate despite technical adjustment, the intraoperative decision to convert to a free nipple graft, accepting the loss of sensation and lactation for the gain of viability, is a legitimate and sometimes necessary application of this assessment [10]. The anatomical, patient-related, technical, and systemic factors influencing NAC perfusion discussed to this point, together with the corresponding preventive measures across the perioperative continuum, are summarized in Table 1.

**Table 1:** Factors influencing NAC perfusion and corresponding preventive measures across the perioperative continuum. The strength of the underlying evidence is deliberately shown as uneven, ranging from randomized trials and meta-analyses to physiologic reasoning. The 'Type of study' column indicates the predominant type of evidence supporting each measure—randomized controlled trial, systematic review/meta-analysis, cohort/observational study, anatomical study, or, where no comparative data exist, physiologic reasoning and clinical observation—as a descriptive indication of the strength of the evidence rather than a formal graded level-of-evidence classification. NAC: nipple-areola complex; RCT: randomized controlled trial; ICG: indocyanine green; NSQIP: National Surgical Quality Improvement Program

Phase	Factor	Fixed/modifiable	Preventive measure	Type of study
Preoperative	Sternal notch-to-nipple distance; breast geometry	Fixed	Risk stratification; weigh pedicle transposition against free nipple grafting	Observational [11]
Preoperative	Resection weight; macromastia	Partly fixed	Pedicle planning; free nipple graft when the pedicle would be excessively long	Observational [2,10]
Preoperative	Obesity	Modifiable (partial)	Weight and glycemic optimization	NSQIP cohort [2]
Preoperative	Smoking	Modifiable	Structured cessation several weeks before surgery	Systematic review/meta-analysis [14,15]
Preoperative	Nutritional status; anemia	Modifiable	Screen and correct deficiency (anemia, protein, iron, vitamin B12, zinc); pertinent in vegetarian/vegan and post-bariatric patients	Review [13]
Intraoperative	Pedicle design and selection	Modifiable (technical)	Reliable medial/superomedial base; avoid excessive length, angulation, and tension	Anatomical/observational [4,5,8,9]
Intraoperative	Hemostasis and hematoma	Modifiable	Meticulous hemostasis; tranexamic acid as adjunct only, not a substitute	Meta-analysis + RCT [18,19]
Intraoperative	Normothermia	Modifiable	Active warming; warmed intravenous and irrigation fluids; temperature monitoring	RCT/review [16,17]
Intraoperative	Perfusion assessment	—	Clinical judgment plus ICG angiography; convert to free nipple graft if marginal	Validation + application [10,22-25]
Systemic	Thromboprophylaxis	Modifiable	Risk-stratified prophylaxis (no direct effect on NAC ischemia)	Model/review [20,21]
Postoperative	Focal external compression	Modifiable	Avoid constrictive bras, bolsters, and bulky dressings	Physiologic reasoning
Postoperative	Surveillance	—	Serial inspection; readiness for early intervention	Clinical observation

Recognition and stepwise salvage of the compromised NAC

When a NAC shows signs of compromise in the early postoperative period, prompt and graded intervention can preserve viability, and distinguishing venous congestion, seen as a dusky, engorged, briskly refilling NAC, from arterial insufficiency, seen as a pale, cool, poorly refilling NAC, guides that intervention. The first maneuver is always to relieve every source of tension and pressure: remove constrictive dressings, release or remove sutures, and, if necessary, reposition the NAC [3]. For incipient arterial compromise, topical nitroglycerin, a cutaneous vasodilator, reduced flap necrosis in a randomized controlled trial in immediate breast reconstruction; although this evidence is extrapolated rather than specific to the NAC, topical nitroglycerin can reasonably be applied to the threatened complex [26]. For refractory venous congestion, medicinal leech therapy is described in the literature to promote decongestion and outflow while venous channels re-form, guided largely by accumulated clinical experience [27]; in the elective aesthetic setting, however, its attendant morbidity - including *Aeromonas* infection, blood loss, and the need for inpatient monitoring - means that early mechanical relief and, when perfusion cannot be restored, timely conversion to a free nipple graft are generally favored, with leech therapy reserved for selected salvageable cases [3]. Hyperbaric oxygen therapy has been used as an adjunct to salvage compromised grafts and flaps by increasing oxygen delivery to marginally perfused tissue, although the supporting evidence is limited and it should be regarded as an adjunct rather than a primary treatment [28]. Comparative series of the different treatments for compromised NAC perfusion after reduction mammaplasty and mastopexy reinforce the value of early, escalating intervention [29]; when the NAC is nonetheless lost, structured management of the established necrosis and, where appropriate, secondary reconstruction minimize the residual deformity [3]. Table 2 summarizes the differential recognition of venous versus arterial compromise and the corresponding stepwise salvage.

**Table 2:** Differential recognition and stepwise salvage of the compromised NAC in the early postoperative period. Venous congestion and arterial insufficiency are distinguished clinically and share the same first maneuver. NAC: nipple-areola complex; RCT: randomized controlled trial

Salvage step	Venous congestion	Arterial insufficiency
Clinical signs	Dusky, engorged; brisk capillary refill	Pale, cool; sluggish capillary refill
1. Relieve tension and pressure	Remove constrictive dressings, release or remove sutures, reposition the NAC [3]	
2. Targeted therapy	Medicinal leech therapy for decongestion and outflow [27]	Topical nitroglycerin, a cutaneous vasodilator (RCT) [26]
3. Adjunct (limited evidence)	Hyperbaric oxygen therapy [28]	
4. If salvage fails	Structured management of established necrosis; secondary reconstruction where appropriate [3]	

A practical prevention framework

Taken together, the evidence supports a prevention framework that spans the entire perioperative continuum rather than any single intervention, as summarized in Table 1. Preoperatively, the surgeon stratifies fixed and modifiable risk and optimizes what can be changed, above all through structured smoking cessation; intraoperatively, sound pedicle selection, atraumatic handling, tension-free inset, meticulous hemostasis, and normothermia protect a perfusion that ICG angiography can confirm, with free nipple grafting held in reserve for the marginal NAC; and postoperatively, avoidance of focal external compression, vigilant serial inspection, and readiness to intervene early complete the sequence.

Limitations

Several limitations temper the strength of these recommendations. First, this is a narrative rather than a systematic review; it was not conducted under a registered protocol with a reproducible search strategy or formal risk-of-bias appraisal, so selection bias in the cited literature cannot be excluded. Second, the evidence base across the individual measures is uneven. Some rest on randomized trials, such as those for topical nitroglycerin and the negative tranexamic acid-hematoma trial, or on robust meta-analyses, such as those for smoking cessation, whereas others, notably the avoidance of external compression from surgical bras, bolsters, and bulky dressings, rest largely on physiologic reasoning and accumulated clinical observation rather than comparative data. Third, much of the perfusion-assessment evidence, including that for ICG angiography, is extrapolated from mastectomy and breast reconstruction rather than derived specifically from aesthetic reduction mammaplasty and mastopexy, in which the pedicle is preserved and the baseline risk profile differs. Fourth, the heterogeneity of outcome definitions and the absence of standardized reporting for partial versus complete NAC necrosis across the source studies limit direct comparison and pooling. These caveats temper, but do not overturn, the central message that most NAC ischemia is anticipated and prevented rather than treated; they also mark out where prospective, reduction-specific data would most strengthen the evidence.

## Conclusions

NAC ischemia, although uncommon in its complete form, remains one of the most consequential complications of reduction mammaplasty and mastopexy. Its prevention rests on the control of perfusion factors that are, to a substantial degree, modifiable. These include thoughtful pedicle selection and atraumatic transposition, attention to smoking, nutritional status, and resection volume, maintenance of normothermia, avoidance of hematoma and external compression, and global perioperative optimization that includes risk-stratified thromboprophylaxis. Attentive perfusion monitoring, supported when available by ICG angiography, enables early recognition and stepwise salvage, from tension relief and topical nitroglycerin to further decongestive and adjunctive measures, before ischemia becomes irreversible. The most effective protection against NAC necrosis is therefore a continuum of careful planning, meticulous technique, and vigilant postoperative surveillance, individualized to each patient and anchored in the best available evidence. Prospective, reduction-specific studies and standardized reporting of NAC outcomes are needed to convert this largely physiologic and observational framework into higher-level evidence.

## References

[1] Fischer JP, Cleveland EC, Shang EK, Nelson JA, Serletti JM (2014). Complications following reduction mammaplasty: a review of 3538 cases from the 2005-2010 NSQIP data sets. Aesthet Surg J.

[2] Nelson JA, Fischer JP, Chung CU (2014). Obesity and early complications following reduction mammaplasty: an analysis of 4545 patients from the 2005-2011 NSQIP datasets. J Plast Surg Hand Surg.

[3] Handel N, Yegiyants S (2016). Managing necrosis of the nipple areolar complex following reduction mammaplasty and mastopexy. Clin Plast Surg.

[4] van Deventer PV (2004). The blood supply to the nipple-areola complex of the human mammary gland. Aesthetic Plast Surg.

[5] O'Dey DM, Prescher A, Pallua N (2007). Vascular reliability of nipple-areola complex-bearing pedicles: an anatomical microdissection study. Plast Reconstr Surg.

[6] Hosny H, Mansour S, El-Essawy NM (2025). Duplex study of the nipple-areola complex blood supply in the female breast. Aesthet Surg J Open Forum.

[7] Robbins TH (1977). A reduction mammaplasty with the areola-nipple based on an inferior dermal pedicle. Plast Reconstr Surg.

[8] van der Meulen JC (1989). Superomedial pedicle technique of reduction mammaplasty. Plast Reconstr Surg.

[9] Brownlee P, Chesire D, Crandall M, Murray J (2017). Superomedial pedicle reduction mammaplasty: increased resection weight does not increase nipple necrosis. J Surg Res.

[10] Sterodimas A, Pineda EF, Meirelles V, Pitanguy I (2008). Maximizing nipple graft survival after performing free nipple-areolar complex reduction mammaplasty. J Plast Reconstr Aesthet Surg.

[11] Park JB, Rahmani B, Adebagbo OD (2024). Impact of sternal notch-to-nipple distance and resection weight asymmetry on complication rates and patient reported outcomes in reduction mammaplasty. J Plast Reconstr Aesthet Surg.

[12] Goldminz D, Bennett RG (1991). Cigarette smoking and flap and full-thickness graft necrosis. Arch Dermatol.

[13] Arnold M, Barbul A (2006). Nutrition and wound healing. Plast Reconstr Surg.

[14] Mills E, Eyawo O, Lockhart I, Kelly S, Wu P, Ebbert JO (2011). Smoking cessation reduces postoperative complications: a systematic review and meta-analysis. Am J Med.

[15] Thomsen T, Tønnesen H, Møller AM (2009). Effect of preoperative smoking cessation interventions on postoperative complications and smoking cessation. Br J Surg.

[16] Kurz A, Sessler DI, Lenhardt R (1996). Perioperative normothermia to reduce the incidence of surgical-wound infection and shorten hospitalization. N Engl J Med.

[17] Sousa Oliveira LA, Da Costa Sacksida Valladão V, Cabette Filho NN, Mendes LH, Zanella FA (2026). Perioperative hypothermia in medium- and large-volume plastic surgery: clinical and surgical implications and practical prevention strategies. Cureus.

[18] Fung E, Montalmant KE, Roth JM (2025). Utility of tranexamic acid in reduction mammaplasty: a systematic review and meta-analysis. Aesthetic Plast Surg.

[19] Yao A, Wang F, Benacquista T (2024). Topical tranexamic acid does not reduce hematoma in reduction mammaplasty: a double-blind randomized controlled trial. Plast Reconstr Surg.

[20] Pannucci CJ (2017). Evidence-based recipes for venous thromboembolism prophylaxis: a practical safety guide. Plast Reconstr Surg.

[21] Sousa Oliveira LA, Helena Mendes L, Cabette Filho NN, da Costa Sacksida Valladão V (2026). Thromboprophylaxis in intermediate and major plastic surgery procedures: a narrative review and proposal of the TROMBO-PLAST integrated decision-making model. Cureus.

[22] Moyer HR, Losken A (2012). Predicting mastectomy skin flap necrosis with indocyanine green angiography: the gray area defined. Plast Reconstr Surg.

[23] Murray JD, Jones GE, Elwood ET, Whitty LA, Garcia C (2010). Fluorescent intraoperative tissue angiography with indocyanine green: evaluation of nipple-areola vascularity during breast reduction surgery. Plast Reconstr Surg.

[24] Li H, Zhang X, Li Z, Lin Y, Mu D (2024). Using laser-assisted indocyanine green angiography to assess nipple-areolar complex survival during reduction mammoplasty. Aesthet Surg J.

[25] Daniels M, Musmann RJ, Andree C (2026). Perfusion control of the nipple-areola complex in reduction plasty for gigantomastia using indocyanine green: a prospective observational study. J Plast Reconstr Aesthet Surg.

[26] Gdalevitch P, Van Laeken N, Bahng S (2015). Effects of nitroglycerin ointment on mastectomy flap necrosis in immediate breast reconstruction: a randomized controlled trial. Plast Reconstr Surg.

[27] Rajic AJ, Deleyiannis FW (2016). Determining the duration of leech therapy in the treatment of acute venous congestion in prefabricated free flaps. Plast Reconstr Surg.

[28] Francis A, Baynosa RC (2017). Hyperbaric oxygen therapy for the compromised graft or flap. Adv Wound Care (New Rochelle).

[29] Reichert R, Weitgasser L, Schoeller T, Wimmer F, Russe E, Mahrhofer M (2025). Compromised nipple-areola complex perfusion after reduction mammaplasty or mastopexy: a retrospective evaluation of different treatments. Aesthetic Plast Surg.

